# Surgical Ablation of Atrial Fibrillation Using Energy Sources

**DOI:** 10.5935/1678-9741.20150078

**Published:** 2015

**Authors:** Alexandre Visconti Brick, Domingo Marcolino Braile

**Affiliations:** 1 Universidade de Brasília (UNB), Brasília, DF, Brazil; 2 Faculdade de Medicina de São José do Rio Preto (FAMERP), São José do Rio Preto, SP, Brazil and Universidade de Campinas (UNICAMP), Campinas, SP, Brazil

**Keywords:** Atrial Fibrillation, Arrhythmias, Cardiac, Ablation Techniques, Energy-Generating Resources, Cardiac Surgical Procedures

## Abstract

Surgical ablation, concomitant with other operations, is an option for treatment in patients with chronic atrial fibrillation. The aim of this study is to present a literature review on surgical ablation of atrial fibrillation in patients undergoing cardiac surgery, considering energy sources and return to sinus rhythm. A comprehensive survey was performed in the literature on surgical ablation of atrial fibrillation considering energy sources, sample size, study type, outcome (early and late), and return to sinus rhythm. Analyzing studies with immediate results (n=5), the percentage of return to sinus rhythm ranged from 73% to 96%, while those with long-term results (n=20) (from 12 months on) ranged from 62% to 97.7%. In both of them, there was subsequent clinical improvement of patients who underwent ablation, regardless of the energy source used. Surgical ablation of atrial fibrillation is essential for the treatment of this arrhythmia. With current technology, it may be minimally invasive, making it mandatory to perform a procedure in an attempt to revert to sinus rhythm in patients requiring heart surgery.

**Table:** 

**Abbreviations, acronyms & symbols**	
AF	= Atrial fibrillation
ISMICS	= International Society of Minimally Invasive Cardiothoracic Surgery
LILACS	= Latin American and Caribbean Health Sciences
Medline	= Medical Literature Analysis and Retrieval System Online
SciELO	= Scientific Electronic Library Online
SICTRA	= Saline-Irrigated Cooled-tip Radiofrequency Ablation

## INTRODUCTION

Atrial fibrillation (AF) is the most common and complex supraventricular arrhythmia with loss of atrial contraction, occurring in about 0.4% of the population and with 10% of the patients being over 60 years old. It is frequently associated with mitral valve disease and it is a constant cause of thromboembolic events, especially cerebrovascular^[1]^.

There are several studies demonstrating that pharmacological means of controlling the rhythm does not reduce morbidity and mortality rate nor does it protect against thromboembolism. The AF that does not respond to medicines has several consequences such as maintaining the irregular rhythm (palpitations and discomfort), loss of atrioventricular synchrony, heart failure, and atrial thrombosis with thromboembolic episodes, being the cause of stroke and pulmonary embolism in 33% of the cases^[2-4]^. Catheter ablation has an outstanding position in the treatment of AF, but surgery is an effective therapeutic method for treating chronic AF in patients who require heart surgery for other reasons.

The most accepted theory to explain the electrophysiological mechanism for maintenance of AF is multiple waves, described by Moe^[5]^ and confirmed by the studies of Alessie et al.^[6]^ and Cox et al.^[7]^. Subsequently, it was shown that AF is initiated by automatic foci with high triggering frequency^[8,9]^. The endocardial mapping revealed that these foci are located in the pulmonary veins and the creation of multi-shaped lesions with radiofrequency energy where foci originate interrupts AF^[10]^. While the cardiac electrical stimulation does not propagate where there is lesion with scarring, requiring normal myocardium to progress, the injury caused by ablation with energy sources interrupts the reentry circuit^[8-10]^.

The International Consensus on Catheter Ablation for Atrial Fibrillation Surgery defined the following indications for surgical ablation of AF^[11]^: 1) symptomatic patients undergoing surgical procedures; 2) selected asymptomatic patients undergoing another surgical procedure in which ablation can be performed with minimal risk; and 3) surgery for primary AF, to be considered for symptomatic patients who opt for surgery in which one or more attempts of catheter ablation have failed or who are not candidates for catheter ablation.

In addition, the Brazilian Guidelines for Treatment of AF indicate the following^[12]^: Class I: patients with symptomatic AF undergoing mitral valve surgery; Class II B: surgery for treatment of AF in patients with symptomatic AF in which catheter ablation cannot be performed or has failed.

The first non-pharmacological treatment of AF includes: 1) electrical cardioversion with a high recurrence rate^[13]^; 2) techniques using intraoperative cryoablation of the bundle of His and the atrioventricular junction^[14]^; 3) isolation of the left atrium^[15]^; 4) catheter ablation of the atrioventricular junction and permanent pacemaker implantation^[9]^; 5) catheter ablation of the bundle of His and definitive pacemaker implant; and 6) "Corridor Operation^[16]^".

Although they regularize heart rate, these procedures maintain the atria, or part of them, fibrillating, thereby not eliminating the risk of complications such as hemodynamic compromise and the occurrence of thromboembolic events.

Based on the electrophysiological mechanisms of AF analyzed in experimental studies, the "Cox operation"has been described, which consists of making incisions and sutures on the atrial wall, enabling the spread of electrical stimulation in the atria within a labyrinth, ordering atrial contraction^[17]^. Changes have been performed to the "Cox Operation", making it simpler and also propitiating the return of sinusal rhythm^[18]^. Studies reporting the success of this technique were published, and it is considered the reference standard for the surgical treatment of AF^[19,20]^, mainly in patients with mitral valve disease^[21]^.

The wide dissection of cardiac structures and the extensive lines of section and suturing of the atrial wall, however, significantly increased operative time as well as cardiopulmonary bypass and aortic clamping time, resulting in higher postoperative morbidity and making the widespread application of the procedure difficult.

In order to increase the applicability and reduce the complexity of the "Cox operation", several technical modifications were introduced, such as the change in the location of the atrial incisions^[22]^; the reduction of section lines and suturing of the atrial wall, known as "Mini Cox^[23]^"; and the unilateral procedure, performed only in the left atrium, called "Cox at Left"^[24]^.

The use of catheter ablation for the treatment of supraventricular arrhythmias stimulated the use of energy sources (cryoablation, radiofrequency, microwave, ultrasound, and laser) to cause linear ablative lesions for endocardial and epicardial applications or to replace the cutting and suturing of the atrial wall^[25-28]^, especially when associated with valvular disease.

The Maze I procedure developed by Cox employed section, suture, and cryoablation^[19]^. Changes in techniques, including the dismissal of cryoablation, have been introduced, such as described by Jazbik et al.^[29]^, Gregori Jr. et al.^[30]^, and Batista et al.^[31]^.

Kalil et al.^[32]^ performing a simplified surgical technique with a single incision around all four pulmonary vein ostia in patients with mitral valve disease, found that this technique was effective in treating AF secondary to mitral valve disease.

Several authors have developed and improved techniques to eliminate chronic AF and return to sinus rhythm in patients undergoing cardiac surgery, especially in patients with mitral valve disease, using various sources of energy capable of causing permanent blocking lines such as microwaves^[33]^, ultrasound^[34]^, cryoablation^[23]^, radiofrequency^[35]^, and laser^[27]^.

Currently, the most widely used energy source is radiofrequency, an alternating current released in the form of an unmodulated, continuous sine wave capable of promoting ablation of the entire tissue. The wave can be either unipolar or bipolar, irrigated or not. The driving of the energy can be measured, demonstrating the transmural lesion, an important factor to eliminate arrhythmia^[36]^. After the use of radiofrequency as the form of energy most commonly applied in catheter ablation, results of experimental studies have been published^[37,38]^ with new sources of energy being used in surgical ablation. Radiofrequency ablation for treatment of AF was described by Hindricks et al.^[35]^.

Regarding cryoablation, there are two energy sources available: nitrogen oxides and argon, the difference being the ability to freeze the tissue. The size and depth of the lesion will depend on factors such as temperature of the ablation catheter, tissue temperature, size of the catheter, duration and number of ablation lines, and the type of source^[20]^. The disadvantage lies in the length of application and its limited use in minimally invasive techniques.

In an experimental study, Manasse et al.^[39]^ used radiofrequency and cryoablation for endocardial and epicardial routes and/or video-assisted thoracoscopy, demonstrating the importance of the pulmonary veins and the presence of transmural lesions created quickly.

Microwave energy uses an electromagnetic field generated by oscillation of the tissue molecules, producing heat with uniform penetration and without burning the surrounding tissues^[28]^. There is also a limitation on its use in minimally invasive surgery, and complications and concerns about possible esophageal perforation.

The laser, despite being a promising form of energy, has been tested only in experimental studies^[27]^.

The effects of ultrasound to treat AF are produced by tissue damage derived from hyperthermia in tissue necrosis, resulting in a transmural lesion that can be used in both endocardial and epicardial applications, in a minimally invasive manner. Considering the experience with the ultrasound scalpel (UltraCision^®^), often used in videolaparoscopic procedures^[40]^ for treating refractory ventricular tachycardia, Brick et al.^[26,41]^ devised a new approach to form lines of lesions, which would determine the partitioning of the left and right atria under shorter surgical and cardiopulmonary bypass time, reflecting positively in postoperative complications.

The aim of this study is to present a literature review on surgical ablation of AF in patients undergoing cardiac surgery considering energy sources and return to sinus rhythm.

## METHODS

This study was based on a review of the literature on chronic AF surgery using energy sources, available in the following databases: Latin American and Caribbean Health Sciences (LILACS), Medical Literature Analysis and Retrieval System Online (Medline), and Scientific Electronic Library Online (SciELO), in Portuguese and English, in the last 20 years. The keywords used included: atrial fibrillation, ablation techniques, energy sources, and results of surgical treatment.

The analysis of the studies was performed descriptively in order to present current knowledge about surgical ablation of AF in patients undergoing cardiac surgery^[42]^.

Experimental, randomized and non-randomized studies were analyzed on the development of surgical ablation of AF with energy sources associated with cardiac surgery.

Catheter ablation studies and surgical treatment of AF alone were excluded.

## RESULTS

In this literature review, we identified 72 studies on evolution and improvement of surgery of arrhythmias, divided into: epidemiology and clinics (n=7), AF theories (n=4), guidelines (n=3), experimental surgery (n=9), pioneering surgery (n=8), section and suturing surgery ("Maze") (n=16), and energy sources surgery (n=25) (Table 1).

**Table 1:** Classification of articles according to clinical and surgical characteristics.

Article	N
Epidemiology and clinical	7
AF Theories	4
Guidelines	3
Surgeries	
Pioneering surgery	8
Experimental surgery	9
Section and suturing surgery ("Maze")	16
Energy sources surgery	25
Total of Selected Studies	72

Nineteen articles were identified from the first surgery to eliminate fibrillation, known as the Maze procedure pioneered by Cox, until the early use of energy sources for ablation of arrhythmia.

Regarding the use of energy sources, Table 2 presents 25 articles focusing on sample size, type of study, results (early and late), and completion (percentage of return to sinus rhythm). Of those, six are randomized trials (484 patients), 17 are non-randomized (1,551 patients), and two are meta-analyzes (74 studies). A total of 19 studies were prospective; 4, retrospective.

**Table 2:** Summary of literature on surgery of chronic atrial fibrillation using energy sources.

Author / Year	Energy Source / Reference	Sample	Type of Study	Outcome*	Conclusion** (%)
	**Radiofrequency**				
Breda et al./2010	53	15	Prospective	Immediate	96.0
Beukema et al./2008	55	258	Retrospective	Late	69.0
Williams et al./2001	56	48	Prospective	Late	81.0
Kottkamp et al./2002	57	70	Prospective	Late	93.0
Benussi et al./2002	58	132	Prospective	Late	76.9
Canale et al./2011	59	53	Retrospective	Late	68.0
Canale et al./2011	60	47	Prospective	Immediate	73.0
Huang et al./2014	61	81	Retrospective	Late	76.0
Dong et al./2013	62	191	Retrospective	Late	79.1
Phan et al./2014	63	62	Meta-analysis randomized controlled trials	Late	67.9
Colafranceschi et al./2009	64	10	Prospective	Immediate	80.0
Abreu Filho et al./2005	65	70	Prospective randomized	Late	79.4
	**Ultrasound**				
Brick et al./2001	68	27	Prospective	Immediate	81.4
Ninet et al./2005	69	103	Prospective	Late	85.0
Lins et al./2010	70	44	Prospective randomized	Late	86.4
Groh et al./2008	71	98	Prospective	Late	84.0
	**Cryoablation**				
Fukada et al./1998	47	29	Prospective	Late	65.5
Lee et al./2001	48	83	Prospective randomized	Late	95.7
Lee et al./2003	49	129	Prospective randomized	Late	97.7
Blomstrom-Lundqvist et al./2007	51	69	Prospective randomized double-blind	Late	73.3
Johansson et al./2012	52	65	Randomized	Late	73.0
	**Microwave**				
Gillinov et al./2002	28	20	Prospective	Immediate	75.0
MacDonald et al./2012	66	12 studies	Meta-analysis	Late	62.0
Lin et al./2011	67	94	Prospective randomized	Late	87.0
	**Electrocautery**				
Gomes et al./2008	45	23	Prospective	Late	76.4

* Late results correspond to those obtained from 6 months of follow-up

** Percentage of return to sinus rhythm

Analyzing studies with immediate results (n=5), the percentage of return to sinus rhythm ranged from 73% to 96%, whereas in the long-term results (n=20) (from 12 months) the range was from 62% to 97.7%. In both, there was subsequent clinical improvement of patients who underwent ablation, regardless of the energy source used.

## DISCUSSION

With research on the origin of AF and with the experience of electrophysiologists and surgeons, new approaches have emerged from the classic Maze surgery (Cox-Maze)^[7,17,18]^, making the extensive manipulation of the atria inconvenient, with peri- and postoperative repercussions. Consequently, there has been a demand for less invasive procedures, such as manipulation only in the pulmonary veins and intraoperative ablation of atrial walls with alternative energy sources (cryoablation, microwave, radiofrequency, laser, and ultrasound), and thoracoscopy procedures using radiofrequency and ultrasound catheters from the epicardium in on-pump surgeries.

Atrial ablation can be performed using the traditional cut and suture technique as well as the simplified technique for pulmonary vein isolation, as demonstrated by Albrecht et al.^[43]^ in a controlled, prospective randomized study.

In a prospective randomized study of patients with rheumatic mitral valve disease with chronic AF, Vasconcelos et al.^[44]^ studied 29 patients (n=13, control group; n=14, treated group) for 11.5 months. The authors concluded that surgical isolation of the posterior wall of the left atrium involving the pulmonary vein ostia is an effective way of treating rheumatic mitral valvular disease in chronic AF.

Gomes et al.^[45]^, using electrocautery in mitral valve surgery, found that this source of energy reversed arrhythmia in a significant number of patients. In the absence of ultrasound equipment, Brick (personal communication) had the opportunity to use electrocautery in a small number of patients, not recommending its routine use due to charring with the release of small emboli inside the atrium.

In the following paragraphs, the energy sources are reviewed with data from the literature.

In regard to cryoablation, Gallagher et al.^[46]^ described its initial use in the treatment of accessory bundles as a method for correcting pre-excitation syndrome. Fukada et al.^[47]^ investigated the indication of "Cox operation"in patients with mitral valve disease and AF using cryoablation to replace some of the atrial incisions. They observed that the ideal cases were patients with disease of non-rheumatic origin, especially those undergoing valve repair.

Lee et al.^[48]^ used cryoablation to replace the section and suture lines, dividing the patients into two groups: in the first one, the lines described in the "Cox operation"were used; in the second, the left and right pulmonary veins were isolated separately. Thus, they studied the improvement of atrial contraction in 83 patients undergoing surgical treatment of AF and other heart diseases. During early evolution and the six-month follow-up, it was demonstrated that the restoration of sinus rhythm and recovery of atrial contraction were significantly more evident in the second group. Later, Lee et al.^[49]^ compared 86 patients with mitral valve disease of rheumatic origin to 43 patients with mitral disease of degenerative etiology and found similar results in both groups after six months, with conversion to sinus rhythm in 95.3% and 97.7% and recovery of atrial contraction in 90.4% and 91.9%, respectively.

Cox et al.^[50]^ described a minimally invasive technique for performing the "Cox operation III", with access through right inframammary thoracotomy of about 7 cm, cannulation of artery, right femoral vein and superior vena cava for installation of cardiopulmonary bypass system and endocardial application of cryoablation to replace the section and suture lines.

In 2007, Blomström-Lundqvist et al.^[51]^ stated the benefit of cryoablation in reversion and maintenance of sinus rhythm in patients undergoing mitral valve surgery. In a prospective multicenter randomized study, they analyzed 69 patients who underwent mitral valve surgery with or without cryoablation in order to assess the efficacy of cryoablation applied to the epicardium of the left atrium in patients undergoing this type of surgery. During follow-up, heart rate was measured at 6 and 12 months, sinus rhythm was regained in 73.3% of the patients who underwent ablation (in both periods) and in 45.7% (6 months) and 42.9% (12 months) of patients who underwent mitral valve surgery alone. There was a significant difference between the groups in the two follow-up periods.

Johansson et al.^[52]^ used epicardial cryoablation in a randomized study with 65 patients undergoing mitral valve surgery, concluding that the ablation group had better results.

This source of energy, originally used by Cox et al.^[50]^, represents an effective and safe therapeutic option. However, the main drawback in minimally invasive surgery (off-pump) is that freezing the blood produces coagulation, resulting in the risk of thromboembolism^[51]^.

Another source of energy, radiofrequency was the first alternative energy source applied in the surgical treatment of AF and it has been widely used. In point-by-point radiofrequency ablation, the irrigated unipolar device produces linear tissue lesions. A bipolar catheter is able to promote ablation of all tissue involved by the electrodes quickly (usually less than 10 seconds). Breda et al.^[53]^ reported the initial assessment of surgical biatrial ablation by radiofrequency. Energy output can be measured during ablation and this can be correlated with proven transmural lesions^[54]^.

In 2008, Beukema et al.^[55]^ published the results of medium- and long-term follow-up after radiofrequency ablation associated with other cardiac surgeries and demonstrated maintenance of sinus rhythm in 69% of the cases treated in the 1-year follow-up, 56% in 3 years, 52% in five years, and 57% in later periods. Treatment with antiarrhythmic drugs was maintained in 64% of the patients who were free of AF; only 1% was under oral anticoagulation regimen.

Williams et al.^[56]^ described the experience of three centers with the use of flexible catheter for applying radiofrequency in pulmonary vein isolation using the endocardial application, similar to the "Cox operation", or separately in the right and left sides, with lesions communicating between the two blocs. The procedure was performed in 48 patients undergoing other associated procedures and, in eight cases, lesions were performed in the right atrium. Results showed survival rates of 87.5% and restoration of sinus rhythm in 81% of the cases at an average follow-up of four months.

Kottkamp et al.^[57]^, through access with anterolateral right mini-thoracotomy and video-assisted cardiopulmonary bypass, performed continuous linear lesions with endocardial application of radiofrequency in the left atrium, involving the mitral annulus and the pulmonary veins after anatomical definition of reentrant circuits with electrophysiological mapping. The procedure was performed in 70 patients with persistent or paroxysmal AF. After six months, 93% of patients were in sinus rhythm; after 12 months, 95% of patients had persistent AF and 97% of patients with paroxysmal AF were in sinus rhythm. Complications from the procedure are a case of esophageal perforation and a case of development of circumflex coronary artery stenosis.

Benussi et al.^[58]^ described the epicardial application of radiofrequency with multipolar catheter for performing lesions in 40 patients with mitral valve disease. The procedure was performed around the right and left pulmonary veins, connecting them to the left atrial appendage. After the left atriotomy, endocardial application joined the lesions of the pulmonary veins with the mitral valve, with the exclusion, in the end, of the left atrial appendage. In the mean follow-up of 11.6 months, 76.9% of the patients had reverted to sinus rhythm, with a significant reduction in the diameter of the left atrium and recovery of right and left atrial contraction. In the medium-term, the results of the experience with radiofrequency ablation for AF in 132 patients were presented, being performed through epicardial application in 107 patients. The lesions in the left atrium were performed prior to cardiopulmonary bypass and used for correction of associated heart diseases. Operative mortality was 0.8%. After three years of development, 77% of the patients were free of AF and 98% were free of stroke, with a survival rate of 94%.

In 2011, Canale et al.^[59,60]^ performed retrospective studies demonstrating that the use of bipolar radiofrequency led to the reversal of arrhythmia in 68% of the patients after 14 months in one of the studies and in 73% of the patients in the period of 7 months according to the other one. The author's experience with the use of bipolar radiofrequency was more efficient than with unipolar. In 2014, Huang et al.^[61]^, after comparing the use of monopolar and bipolar radiofrequency, concluded that both are effective for the treatment of chronic AF, however, ablation with bipolar radiofrequency is more convenient.

Dong et al.^[62]^, in 2013, analyzed concomitant valve replacement and radiofrequency ablation in 191 patients with rheumatic disease; 158 patients were followed-up for a year and sinus rhythm was maintained in 79.11%.

In 2014, Phan et al.^[63]^, after performing cumulative metaanalysis of randomized clinical trials of surgeries with and without AF ablation in six databases, identified sixteen randomized trials and concluded that surgical ablation concomitant with cardiac surgery was effective and safe to restore sinus rhythm, after 12 months of follow-up.

Colafranceschi et al.^[64]^ performed video-assisted thoracoscopic surgery in 2009, concluding that it is safe and reproducible, especially in patients with paroxysmal fibrillation refractory to drug therapy who did not require concomitant surgery. This technique opens new paths for patients who have not responded adequately to catheter treatment.

In Brazil, Brick et al.^[26]^ started to experience with a unipolar radiofrequency catheter while performing point-by-point ablation of the left atrium. The development of bipolar ablation devices with irrigated catheter contributed to the technical improvement of the procedure, leading to shorter operative time and postoperative results showing a satisfactory success rate of 96% of sinus rhythm reversal^[53]^.

Abreu Filho et al.^[65]^ evaluated 70 patients with permanent AF and rheumatic mitral valve disease. These patients were randomly assigned to undergo either a modified Maze III procedure using Saline-Irrigated Cooled-tip Radiofrequency Ablation (SICTRA) associated with mitral valve surgery (group A) or mitral valve surgery alone (group B). The cumulative rates of sinus rhythm were 79.4% in group A and 26.9% in group B (*P*=0.001). They concluded that SICTRA is effective for treating permanent AF associated with rheumatic mitral valve disease.

Using microwave as an energy source, Gillinov et al.^[28]^ described their experience with ablation in 10 patients undergoing surgery for mitral valve and pulmonary vein isolation for epicardial application. After opening the left atrium, the lesions were observed from the endocardial surface, proving to be transmural.

In 2012, MacDonald et al.^[66]^ concluded that microwave energy concomitant with surgery was not as effective when compared to radiofrequency ablation in long-term outcome assessment.

Lin et al.^[67]^, in 2011, compared the use of microwave in 94 patients to the use of bipolar radiofrequency in 93 patients undergoing valve surgery, for three months, concluding that radiofrequency ablation is superior to ablation with microwave.

The use of ultrasound in the treatment of AF promotes tissue damage by hyperthermia. This technology is attracting interest because it allows for ablation in a less invasive manner, without damaging adjacent structures. Brick et al.^[68]^ began their experience with ultrasound using the unipolar endocardial catheter ablation and three patients underwent surgery with reversion to sinus rhythm.

Execution of intraoperative ablation using this technique, in addition to facilitating and reducing the operative time, allowed for greater understanding of the role of the left atrium and pulmonary veins in chronic fibrillation^[26,37]^.

Additional procedures were performed, such as exclusion of the right and left atrial appendages; reduction of the left atrium, where necessary; and ablation of the right atrium to eliminate the possibility of atrial flutter.

Ninet et al.^[69]^, in a multicenter study, prospectively analyzed 103 patients between September 2002 and February 2004, using ultrasound for epicardial ablation of AF. Ablation was performed through epicardial application using a high-frequency ultrasound, EpiCor^®^ (St Jude Medical Inc). Analysis showed that, after six months, 85% of patients were in sinus rhythm. They demonstrated the advantage of using the energy of ultrasound, creating a transmural lesion around the left atrium without the use of cardiopulmonary bypass.

Lins et al.^[70]^ showed an improvement in functional class in the treated group *versus* the control group in a comparative study of patients with mitral valve disease, using ultrasound scalpel (UltraCision^®^). The groups presented homogeneous preoperative characteristics and no significant difference considering cardiopulmonary bypass times, anoxia and postoperative intensive care unit stay, which leads to the conclusion that the use of ultrasonic scalpel for AF ablation does not act to worsen patient outcomes. Comparing patients who underwent ultrasound ablation with those who received no recent ultrasound ablation, no more complications or deaths in recent or late postoperative were found. The results observed in this study show that the ultrasound ablation technique can be applied to patients who have surgical indication for mitral valve disease correction.

In addition to the endocardial application, ultrasound with other devices such as the EpiCor^®^ can be used in off-pump surgery, through epicardial application in patients with isolated fibrillation, as well as in patients with ischemic heart disease undergoing coronary artery bypass graft surgery^[71]^.

The objective of the 2009 Consensus Conference of the International Society of Minimally Invasive Cardiothoracic Surgery (ISMICS)^[72]^ was to determine whether surgical ablation of AF during associated cardiac procedures improved postoperative clinical outcomes. The group involved in the study analyzed the best available evidence, with systematic data review, including randomized and non-randomized controlled studies, always in descending order of importance. A systematic review and meta-analysis identified 10 randomized trials (650 patients) and 23 non-randomized (3997 patients); the great majority was published in English and performed in the United States.

The authors of the consensus defined the following recommendation: in patients with persistent and permanent AF, surgical ablation is recommended to increase the incidence of sinus rhythm in the short- and long-term (Class 1, Level A); reduce the risk of stroke and thromboembolic events (Class 2a, level A); increase exercise tolerance and improve ventricular function (Class 2a, level A); and increase survival (Class 2a, level B)^[72]^.

## CONCLUSION

Evolving from the classical labyrinth surgery (Cox-Maze), changes in demand for less invasive procedures have occurred with the use of alternative sources of energy.

The results of the surgical ablation of AF in patients undergoing cardiac surgery depend on the energy source used; the lesion produced is transmural and applied in both atria.

We conclude that surgical ablation of AF is essential in the treatment of this arrhythmia. With current technology, it may be minimally invasive, making it mandatory to perform a procedure in an attempt to revert to sinus rhythm in patients requiring heart surgery.

Situations involving primary fibrillation surgery indication should include a multidisciplinary team approach involving cardiologists, electrophysiologists and surgeons to make the correct choice of patients and the most appropriate procedure.

**Table:** 

**Authors' roles & responsibilities**	
AVB	Study design; writing of the manuscript or critical review of its content; final approval of the manuscript
DMB	Study design; writing of the manuscript or critical review of its content; final approval of the manuscript
